# Novel Vitamin D Analogs for Prostate Cancer Therapy

**DOI:** 10.5402/2011/301490

**Published:** 2011-09-19

**Authors:** Tai C. Chen, Atsushi Kittaka

**Affiliations:** ^1^Boston University School of Medicine, Room M-1022, 715 Albany Street, Boston, MA 02118, USA; ^2^Faculty of Pharmaceutical Sciences, Teikyo University, Midori-ku, Sagamihara, Kanagawa 252-5195, Japan

## Abstract

Prostate cells contain specific receptors for 1*α*,25-dihydroxyvitamin D [1*α*,25(OH)_2_D] or calcitriol, the active form of vitamin D. 1*α*,25(OH)_2_D is known to inhibit the proliferation and invasiveness of prostate cancer cells. These findings support the use of 1*α*,25(OH)_2_D for prostate cancer therapy. However, 1*α*,25(OH)_2_D can cause hypercalcemia, analogs of 1*α*,25(OH)_2_D that are less calcemic but exhibit potent antiproliferative activity would be attractive as therapeutic agents. To accomplish these goals, different strategies, based on metabolism, molecular mechanism of actions, and structural modeling, have been taken to modify the structure of vitamin D molecule with the aims to improve the efficacy and decrease the toxicity of vitamin D to treat different diseases. During the past four decades, over 3,000 analogs have been synthesized. In this paper, we discuss the development and the biological analysis of a unique class of vitamin D analogs with a substitution at the carbon 2 of 19-nor-1*α*,25(OH)_2_D_3_ molecule for potential application to the prevention and treatment of prostate cancer as well as other cancers.

## 1. Introduction

Prostate cancer is the most prevalent nonskin cancer among older men, with 190,000 new cases in 2010, and the second leading cause of cancer deaths among US men after lung cancer with approximately 30,000 deaths projected in 2011 [1]. Androgen deprivation is a common practice for tumors that are ineligible for or fail to respond to surgery or radiation therapy. Although the majority of men respond to androgen deprivation, the median duration of response is only about 2 years [2]. Great efforts have been made to develop new therapies to prolong the survival of patients with prostate cancer that fails to response to androgen deprivation therapy, such as docetaxel- or carbazitaxel-based chemotherapy, immunotherapy with sipuleucel-T, or abiraterone acetate. However, their effectiveness is limited to only a few months gain in survival [3].

**Figure 1:**
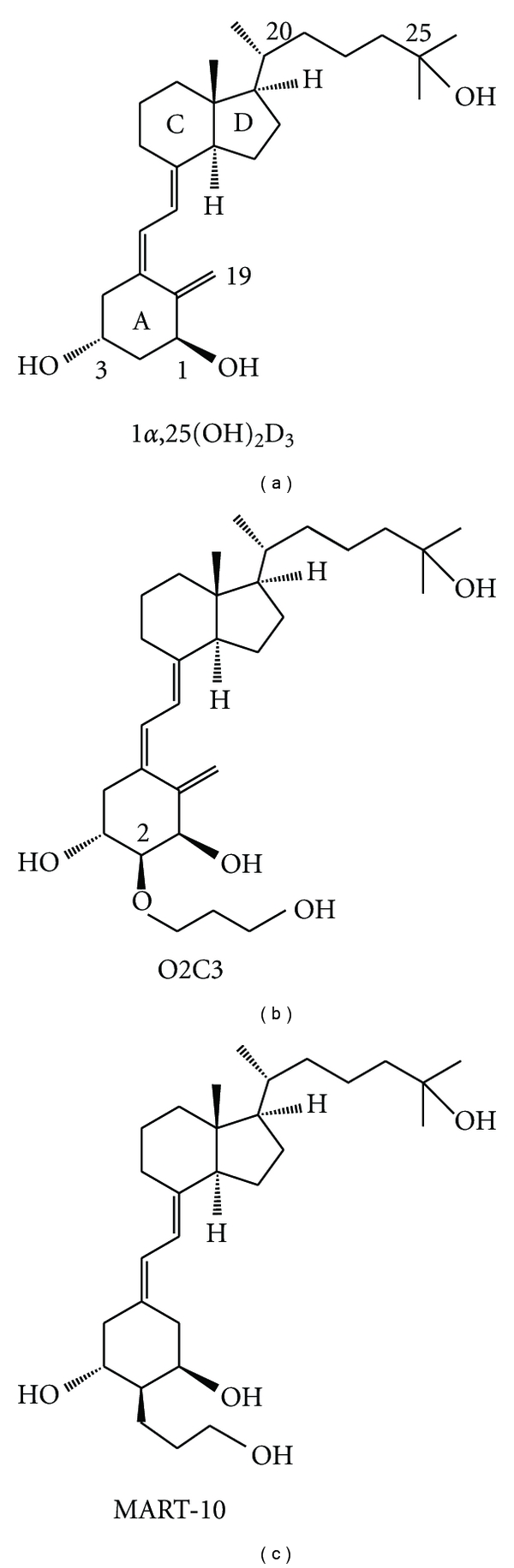
Structures of 1*α*,25(OH)_2_D_3_, 2*α*-3-hydroxypropoxy-1*α*,25(OH)_2_D_3_ (O2C3) and 19-nor-2*α*-3-hydroxypropyl-1*α*,25(OH)_2_D_3_ (MART-10).

It is now well established that the growth of prostate cells is regulated not only by androgens but also by vitamin D. Human prostate cells express vitamin D receptor (VDR) for 1*α*,25-dihydroxyvitamin D [1*α*,25(OH)_2_D] (Figure 1), the active form of vitamin D [4]. Numerous reports have demonstrated that 1*α*,25(OH)_2_D stimulates differentiation and inhibits the proliferation, invasiveness, and metastasis of prostate cancer cells [4]. These findings strongly support the use of vitamin D-based therapies for prostate cancer treatment once androgen deprivation has failed. However, the results of early clinical trials using 1*α*,25(OH)_2_D_3_ (Calcitriol) indicated that the hormone caused serious hypercalcemic and hypercalciuric side effects [5, 6]. Therefore, analogs of 1*α*,25(OH)_2_D with less calcemic activity but with potent antiproliferative activity would be attractive agents.

## 2. History of the Synthesis of Vitamin D Analogs

Vitamin D was discovered a century ago because of an epidemic of a childhood bone disease, rickets [7]. It was concluded that the primary function of vitamin D in humans was to enhance the intestinal absorption of calcium and phosphate, two major ingredients of bones, to maintain their serum concentrations at a sufficient level to facilitate bone calcification and other cellular functions [8]. It was later realized that vitamin D itself was not active and required a series of hydroxylation steps first in the liver and then in the kidneys to form 25-hydroxyvitamin D [25(OH)D] and 1*α*,25(OH)_2_D, respectively, before it became active [8]. Although vitamin D was widely used in the 1940s to treat various forms of skin diseases, such as lupus vulgaris [9], cutaneous tuberculosis [10], and psoriasis [11], it was not until late 1970s that vitamin D receptors were shown to be present in many cells and tissues which were not known to be associated with calcium and phosphorus homeostasis [12]. Subsequently, it was shown that 1*α*,25(OH)_2_D was capable of inhibiting the growth of various types of cancer cells [13] and promoting the differentiation of promyelocytic leukemia cells (HL-60) to form mature macrophages [14]. The *in vitro* cell culture studies were followed by animal studies using xenograft mouse model and chemically induced cancer model to demonstrate the effectiveness of 1*α*,25(OH)_2_D in inhibiting the tumor cell growth [4, 15, 16]. However, 1*α*,25(OH)_2_D at high doses which were usually required to inhibit tumor cell growth *in vivo* animal models also raised serum calcium level and decreased the body weight of the animals [16]. In human clinical trials, hypercalcemia and hypercalciuria were also found to be the major side effects of 1*α*,25(OH)_2_D when it was administered systemically [5, 6]. To overcome these drawbacks, attempts have been made to synthesize vitamin D analogs that retain most of the nonclassical activities of 1*α*,25(OH)_2_D but have much lower calcemic activity *in vivo*. Several synthetic vitamin D analogs have been demonstrated to exert promising anticancer effects with reduced calcemic consequence and even greater antiproliferative activity than 1*α*,25(OH)_2_D [16]. Among those vitamin D analogs, Seocalcitol (EB1089, Leo Pharmaceutical Products) is one of the most studied synthetic analogs [17–19]. A considerable number of *in vitro* and* in vivo *studies have been carried out with EB1089 and show that the analog is more potent than 1*α*,25(OH)_2_D with respect to the regulation of cancer cell growth and differentiation, and the effect of EB1089 on calcium metabolism *in vivo *is approximately 50% less than that of 1*α*,25(OH)_2_D [16]. The anticancer effects of EB1089 were also demonstrated *in vivo *in a rat model of mammary gland carcinoma without inducing hypercalcemia [17, 18]. Similar effects were seen in an *in vivo *prostate cancer study where EB1089 inhibited prostate cancer cell proliferation and reduced tumorigenesis as well as tumor metastases [19]. Clinical trials with 1*α*-hydroxyvitamin D_2_ (Hectorol) marketed by Genzyme have been conducted in hormone refractory prostate cancer patients [20, 21]. In a phase I trial, the authors reported that Hectorol was well tolerated with the main toxicities being hypercalcemia and renal insufficiency, and the side effects were reversible with drug discontinuation [20]. In a follow-up phase II study, they observed disease stability >6 months in 30% of the patients and the median survival of 21 months, which is higher than the 17.7 months predicted by the survival nomogram for that patient group [21]. Although the results are less than conclusive, the encouraging findings do warrant further studies with vitamin D analogs. Other vitamin D analogs or structural VDR activators, such as Maxacalcitol (OCT) (Chugai Pharmaceutical Co. Ltd.) [22], 16-ene analogs (Hoffmann LaRoche, Inc.) [23], 19-nor analogs (Hoffmann LaRoche, Inc.) [24], 1*α*-hydroxyvitamin D_5_ [25], and LG190119 (Ligand Pharmaceuticals Inc.) [26], have been developed and tested in preclinical studies. These compounds may have promise as therapeutic agents for cancer and other diseases, with fewer side effects than 1*α*,25(OH)_2_D_3_ and 1*α*-hydroxyvitamin D_2_. Other vitamin D analogs which have shown some promising *in vitro* biological activities include C-20 cyclopropylcalcitriol [27], elocalcitol [28], Gemini vitamin D analogs [29].

## 3. Development of the Less Calcemic 19-Norvitamin D Analogs

19-Norvitamin D compounds in which the ring A methylene group on C-19 is replaced with two hydrogen atoms were known to be in existence in 1983 when 19-nor-10-ketovitamin D derivatives were first isolated and identified from a mixture of vitamin D or 25(OH)D solution incubated with bovine rumen microbes [30]. Later, 19-nor-1*α*,25(OH)_2_D_3_ was synthesized by Perlman et al. to study the structure-activity relationship of 1*α*,25(OH)_2_D_3_ molecule [31]. They reported that this analog induced the differentiation of human leukemia HL-60 cells similar to 1*α*,25(OH)_2_D_3_ with little or no calcemic activity [31]. The findings led to the synthesis of 19-nor-1*α*,25(OH)_2_D_2_ and the evaluation of its activity by Slatopolsky et al. [32]. Both 19-nor-1*α*,25(OH)_2_D_2_ and 19-nor-1*α*,25(OH)_2_D_3_ have potency similar to 1*α*,25(OH)_2_D_3_ in inducing CYP24A1 promoter activity in a transcription assay and in suppressing parathyroid hormone secretion in hemodialysis patients with secondary hyperparathyroidism, without inducing hypercalcemia or hyperphosphatemia [30–33]. Today, 19-nor-1*α*,25(OH)_2_D_2_, also called Zemplar or paricalcitol, is an FDA-approved drug for the treatment of secondary hyperparathyroidism. Subsequently, different modifications of the A-ring, including the synthesis of C-2 modified 19-norvitamin D compounds, were accomplished by a significant number of synthetic organic chemists [34–41]. 

The first synthesis of 19-norvitamin D reported by DeLuca's group in 1990 is a direct synthesis starting from 25-hydroxyvitamin D_3_ [31]. Subsequently, a convergent synthetic route was described by the same group, and the method has become one of the standard ones for the synthesis of new 19-norvitamin D analogs [42]. The convergent method consists of a coupling reaction between an A-ring phosphine oxide with a C1–C7 carbon unit and an 8-keto-CD-ring with a C17 side chain such as 25-hydroxy Grundmann's ketone. 

**Figure 2:**
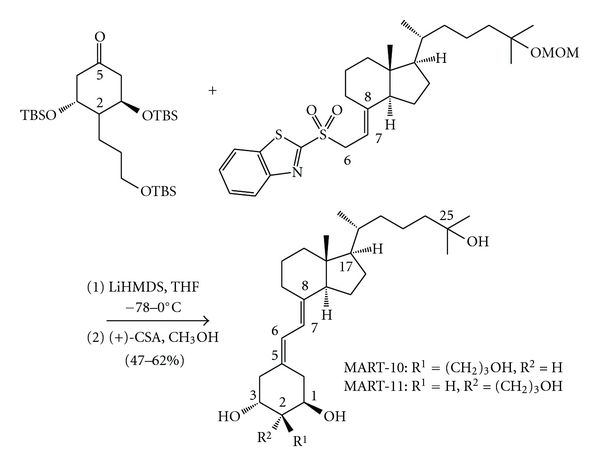
Julia coupling reaction between the A-ring C5 and the CD-ring C6 positions. TBS: *tert*-butyldimethylsilyl group as a protecting group, MOM: methoxymethyl group as a protecting group, LiHMDS: lithium hexamethyldisilazide, THF: tetrahydrofuran, (+)-CSA: (+)-10-camphorsulfonic acid. The structures are written with steroidal numbering.

During the past decade, systematic synthesis of vitamin D_3_ analogs with C2-modification has been attempted, and a number of C2-modified analogs with a greater VDR agonistic activity than 1*α*,25(OH)_2_D_3_ have been successfully synthesized [34, 35, 42–44]. For example, a substitution with 2*α*-methyl, 2*α*-(3-hydroxypropyl), or 2*α*-(3-hydroxypropoxy) group increased its binding affinity for the VDR two- to fourfold compared to 1*α*,25(OH)_2_D_3 _[43–45]. Similarly, several highly potent VDR antagonists, which belong to a series of TEI-9647 analogs with C2*α* functionalization as well as the 24-alkyl modification on the lactone ring, have been synthesized [46]. The mechanism of the enhanced C2*α*-effects on VDR binding affinity has been revealed by an X-ray co-crystallographic analysis of the VDR-ligand complexes [47]. The study shows that the terminal hydroxy group of 2*α*-(3-hydroxypropyl) or 2*α*-(3-hydroxypropoxy) substituent plays an important role in expelling the water molecules in the ligand binding domain of the VDR to form hydrogen bonds with arginine-274 residue of the VDR molecule to stabilize the VDR-ligand complex [47]. Knowing the advantage of modifying 1*α*,25(OH)_2_D_3_ molecule with “2-substitution” to enhance VDR binding affinity and “19-demethylenation” to eliminate calcemic potential, we, therefore, set forth to synthesize C2-substituted 19-nor-1*α*,25(OH)_2_D_3_ analogs using the convergent synthetic approach developed by DeLuca's laboratory [42]. However, during the synthesis of 19-nor-2*α*-3-hydroxypropyl-1*α*,25(OH)_2_D_3_ (MART-10) and 19-nor-2*β*-3-hydroxypropyl-1*α*,25(OH)_2_D_3_ (MART-11), we quickly realized that the typical coupling reaction between the 2-substituted 19-nor-A-ring part and the 8-keto-CD-ring part, that is, C7-C8 connection based on the Horner-Wadsworth-Emmons reaction, was problematic because of the large 1,2-steric repulsion between 1*α*-siloxy and the phenyl groups on phosphorus atom present in the oxaphosphetane transition state [34]. We decided to connect one of the double bonds of the target diene of MART-10/MART-11 between the C5 (A-ring) and C6 (two carbons elongated CD-ring from the 8-keto group) positions using the Julia coupling approach, and the reactions turned out to be successful (Figure 2). Finally, the target products were separated using a reversed phase HPLC to obtain two diastereomers, MART-10 (2*α*-form) and MART-11 (2*β*-form) [48]. 

The MART-10 and MART-11 obtained were then studied for their VDR binding property using a calf thymus vitamin D receptor preparation and HL-60 differentiation potency. We found that MART-10 and MART-11 had a binding affinity equal to 100% and 3% of the parent hormone 1*α*,25(OH)_2_D_3_, respectively. However, we observed a 36-fold and 7-fold greater activity in the induction of HL-60 cell differentiation by MART-10 and MART-11, respectively, than by 1*α*,25(OH)_2_D_3_ [35, 49]. The discrepancy between VDR binding and differentiation activity can be explained at least in part by a greater ability in recruiting coactivators, such as hTIF-2 and hSRC-1, by MART-10 and MART-11 than by 1*α*,25(OH)_2_D_3_ as determined by a high-throughput screening method developed in our laboratory to study the interaction between human VDR and cofactors [50].

## 4. The Antiproliferative Activity of 19-Norvitamin D Analogs in Prostate Cells

**Figure 3:**
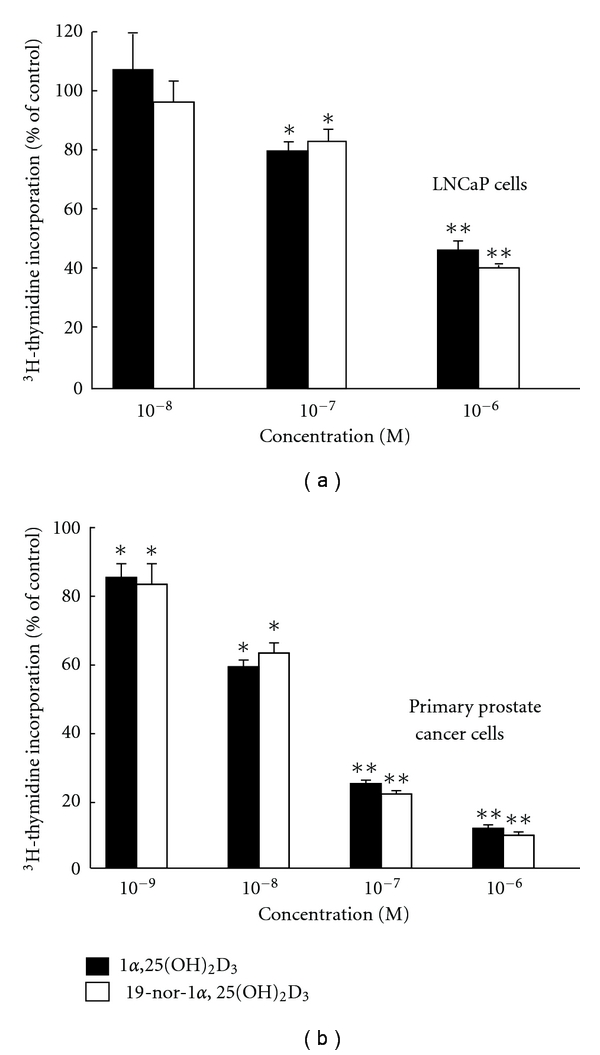
Comparison of the effect of 1*α*,25(OH)_2_D_3_ and 19-nor-1*α*,25(OH)_2_D_3_ on the ^3^H-thymidine incorporation in (a) LNCaP cells and (b) the primary cultures of prostate cancer cells. Results are presented as the means ± SD of 5–8 determinations. There is no difference between 1*α*,25(OH)_2_D_3_ and 19-nor-1*α*,25(OH)_2_D_3_ at any dose levels examined. *P < 0.05, **P < 0.001 versus controls.

The antiproliferative activity of 19-norvitamin D analogs was first studied in LNCaP prostate cancer cells and in primary cultures of prostate cancer cells using 19-nor-1*α*,25(OH)_2_D_2_ and 19-nor-1*α*,25(OH)_2_D_3_ [50, 51]. It was reported that 19-nor-1*α*,25(OH)_2_D_2_ had antiproliferative activity comparable to 1*α*,25(OH)_2_D_3_, as determined by ^3^H-thymidine incorporation [51]. Similarly, 19-nor-1*α*,25(OH)_2_D_3_ was found to be equipotent to 1*α*,25(OH)_2_D_3_ in the primary cultures of prostate cancer cells and LNCaP prostate cancer cells (Figure 3) [52]. After we obtained a series of 19-nor-1*α*,25(OH)_2_D_3_ analogs modified at C-2 position with different hydrocarbon moieties, we began to study their antiproliferative activity in PZ-HPV-7 prostate cells, a cell line derived from the epithelial zone of a normal prostate and obtained from ATCC. Among them, 19-nor-2*α*-3-hydroxypropyl-1*α*,25(OH)_2_D_3_ (MART-10) and 19-nor-2*β*-3-hydroxypropyl-1*α*,25(OH)_2_D_3_ (MART-11) were found to be about 500- to 1,000-fold more active than 1*α*,25(OH)_2_D_3_ [53].

**Figure 4:**
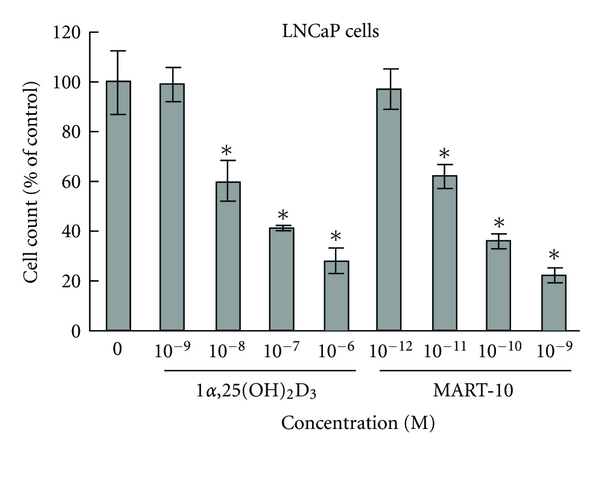
The dose-dependent effects of 1*α*,25(OH)_2_D_3_ and MART-10 on LNCaP cell proliferation. LNCaP cells were treated with ethanol vehicle or the indicated concentrations of 1*α*,25(OH)_2_D_3_ and MART-10 for one week and then trypsinized and cell counted with hemocytometer. The results are expressed as the percent of control of the means ± SD of 3 determinations from a representative experiment. The experiment was repeated 3 times with similar results. *P < 0.05 compared to the previous doses.

Comparison of the inhibitory effect of MART-10 with 1*α*,25(OH)_2_D_3_ on the cellular proliferation was then carried out in androgen-dependent LNCaP and androgen-independent PC-3 prostate cancer cells by hemocytometer cell counting. Similar to the findings using PZ-HPV-7 cells, MART-10 is about 1,000-fold more active than 1*α*,25(OH)_2_D_3_ in inhibiting LNCaP (Figure 4) [54] and PC-3 prostate cancer cell proliferation (D. Iglesias-Gato et al., unpublished data).

## 5. Metabolism of MART-10 by 24-Hydroxylase (24-OHase or CYP24A1)

CYP24A1 is one of the three major enzymes involved in the metabolism of vitamin D endocrine system. The gene encoding this enzyme is highly inducible by 1*α*,25(OH)_2_D_3_ or its analogs, and, therefore, the induction of this gene has been used as an index for the biological potency of new analogs [55, 56]. Most importantly, CYP24A1 serves as a principle mechanism to terminate the biological actions of 1*α*,25(OH)_2_D_3_ or its analogs through 24-hydroxylation-dependent catabolic pathway [57]. Because of this important role, specific inhibitors of CYP24A1 have been developed and used to enhance and prolong the actions of the natural hormone [57]. Alternatively, analogs which are more resistant to the catabolic degradation of CYP24A1 will have longer half-life and potentially more bioavailablity than 1*α*,25(OH)_2_D_3_. 

In a series of experiments comparing the expression of *CYP24A1* in response to 1*α*,25(OH)_2_D_3_ and MART-10 treatment in LNCaP and PC-3 prostate cancer cells, we observed that MART-10 was capable of inducing *CYP24A1* expression at a lower concentration and to a greater extent [54] and with a longer duration than 1*α*,25(OH)_2_D_3_ (D. Iglesias-Gato et al., unpublished observation). The longer duration suggests that MART-10 is more resistant to CYP24A1 degradation. To find out whether this was the case, we then used a cell-free CYP24A1 reconstituted system [54] to determine the *k*_cat_/*K*_*m*_ value, an indicator of enzyme susceptibility, of MART-10 or 2*α*-(3-hydroxypropoxy)-1*α*,25(OH)_2_D_3_ (O2C3) (Figure 1) used as a substrate. We found that MART-10 had a *k*_cat_/*K*_*m*_ of 0.33 which is about 1/10 of what was found with O2C3 (*k*_cat_/*K*_*m*_ = 3.0). Since the *k*_cat_/*K*_*m*_ for O2C3 is about 1/50 of 1*α*,25(OH)_2_D_3_ [58], the *k*_cat_/*K*_*m*_ for MART-10 is about 1/500 of 1*α*,25(OH)_2_D_3_. The *k*_cat_/*K*_*m*_ data suggest that the addition of 3-hydroxypropyl group at carbon 2 in the MART-10 molecule may hinder the contact of the side-chain of MART-10 molecule to the heme group of CYP24A1 and results in a very poor 24-hydroxylation and, in turn, much less degradation of MART-10 by CYP24A1. The conclusion is supported by a docking model of MART-10 sit inside the substrate-binding pocket of the human CYP24A1 (Personal communication with Drs. Yamamoto and Sakaki).

## 6. MART-10 Is a More Potent Inhibitor of Cancer Cell Invasion

**Figure 5:**
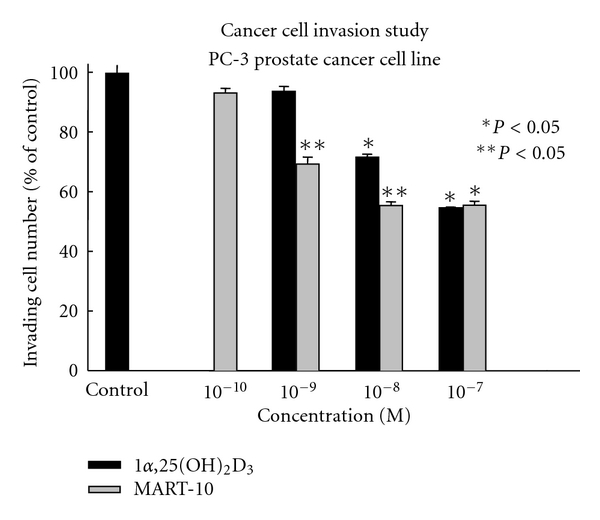
Effect of 1*α*,25(OH)_2_D_3_, and 19-nor-2*α*-3-hydroxypropyl-1*α*,25(OH)_2_D_3_ (MART-10) on the invasion of PC-3 cells. The results are presented as the means ± SD of 3 determinations. *Comparison between control and 1*α*,25(OH)_2_D_3_ or MART-10; **Comparison between 1*α*,25(OH)_2_D_3_ and MART-10.

In addition to its higher activity in inhibiting prostate cancer cell proliferation, MART-10 is about 10-fold more active than 1*α*,25(OH)_2_D_3_ in inhibiting PC-3 cell invasion (Figure 5) [53]. Similar results were obtained with 19-nor-2*β*-3-hydroxypropyl-1*α*,25(OH)_2_D_3_ (unpublished observation). MART-10 also exhibited a greater downregulation of matrix metalloproteinase-9 (MMP-9) expression at both the transcriptional and translational levels (D. Iglesias-Gato et al., unpublished observation). Since MMP-9 is an enzyme involved in the cell invasion pathway [59, 60], the greater downregulation of MMP-9 activity may be responsible for the more potent anti-invasion effect observed in the presence of MART-10. In turn, the greater effect on MMP-9 gene expression and the expression of *CYP24A1* and genes involved in cell proliferation may be due to its more profound VDR transactivation activity in prostate cancer cells [54, 61].

## 7. MART-10 Binds to Vitamin-D-Binding Protein (DBP) with a Lower Affinity Than 1*α*,25(OH)_2_D_3_

**Figure 6:**
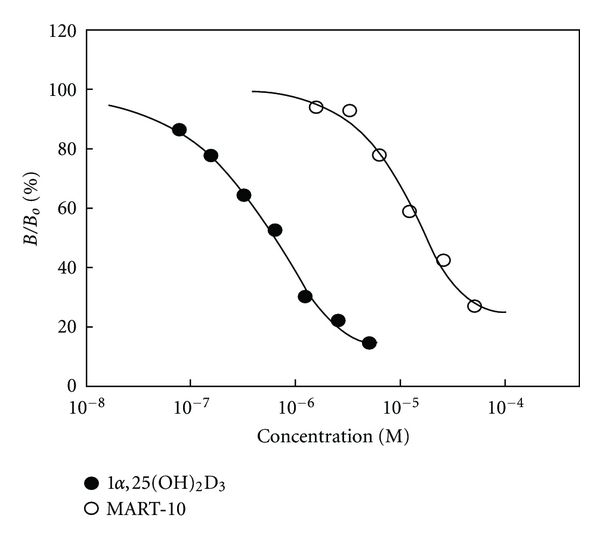
Binding of MART-10 and 1*α*,25(OH)_2_D_3_ to vitamin D binding protein (DBP). The binding affinity of 1*α*,25(OH)_2_D_3_ and MART-10 to vitamin-D-binding protein (DBP) was determined by the displacement of [^3^H]-25(OH)D_3_ from rat serum DBP by indicated concentrations of MART-10 and 1*α*,25(OH)_2_D_3_. The results are expressed as the percentage of displaced [^3^H]-25(OH)D_3_ (B) over total specific bound of [^3^H]-25(OH)D_3_ (Bo).

The bioavailability of MART-10 in circulation was examined by measuring its binding affinity to serum DBP (Kd, defined as the concentration of 1*α*,25(OH)_2_D_3_ or MART-10 at which a 50% reduction in [^3^H]-25(OH)D_3_ binding to DBP was observed). The Kds for MART-10 and 1*α*,25(OH)_2_D_3_ are 17.5 *μ*M and 0.67 *μ*M, respectively, indicating that the binding affinity of MART-10 for DBP is about 25-fold less than that for 1*α*,25(OH)_2_D_3_ (Figure 6) [54]. The lower DBP binding affinity for MART-10 will allow more MART-10 to be translocated into the target cells, including the prostate.

## 8. Conclusion

**Figure 7:**
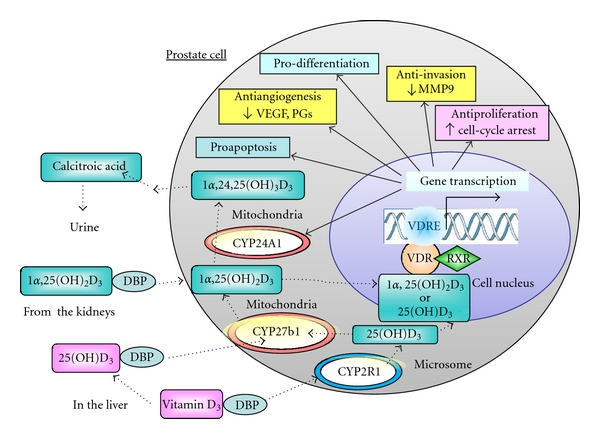
Metabolism and the nonclassical actions of vitamin D in prostate cells. Prostate cells express vitamin D 25-hydroxylase (25-OHase, or CYP2R1, a microsomal enzyme), 1*α*-OHase (or CYP27B1, a mitochondrial enzyme), and 24-OHase (or CYP24A1, a mitochondrial enzyme) and, therefore, are capable of synthesizing 1*α*,25(OH)_2_D_3_ from vitamin D_3_. Binding of 1*α*,25(OH)_2_D_3_ or 25(OH)D_3_ to the vitamin D receptor (VDR) causes the VDR to heterodimerize with the retinoid X receptor (RXR). The VDR-RXR heterodimer binds to specific vitamin D response elements in the promoter region of vitamin-D-responsive genes and induces gene transcription. The gene products include proteins involved in its own metabolism (CYP24A1), cell-cycle arrest, apoptosis, differentiation, anti-invasion, antiangiogenesis, and many other actions.

Vitamin D has been discovered as an antirachitic agent for almost a century. For more than half a century since its discovery, vitamin D was believed to be involved only in calcium and phosphate homeostasis. The realization that vitamin D (vitamin D_2_ and vitamin D_3_) itself was not active and required two successive hydroxylation steps to produce its active form, 1*α*,25(OH)_2_D_3_, led to the finding in 1979 that VDR was present in many tissues not related to calcium and phosphate metabolism. Subsequently, many non-classical actions of 1*α*,25(OH)_2_D_3_ were revealed, including antiproliferation, anti-invasion, proapoptosis, prodifferentiation, immune regulation, and so forth, (Figure 7). Although 1*α*,25(OH)_2_D_3_ exhibited potent antitumor effects on prostate cancer models, hypercalcemia and hypercalciuria side effects were quickly realized in animal models and human clinical trials. The lethal side effects, thus, limit the application of 1*α*,25(OH)_2_D_3_ clinically. Consequently, several thousand vitamin D analogs were synthesized with an intention to eliminate or lessen the side effects and at the same time to enhance their antitumor activity. So far, none of the analogs have shown clinically satisfactory results. In this paper we describe the synthesis of a novel analog of 1*α*,25(OH)_2_D_3_, called MART-10 and present *in vitro* data using normal, androgen-dependent LNCaP, and androgen-independent PC-3 cell culture models. Comparing to 1*α*,25(OH)_2_D_3_, MART-10 is 10 times more active in stimulating VDR transactivation in LNCaP cells, about 500- to 1000-fold more active in inhibiting the proliferation of these three types of prostate cells, 10 times more potent in inhibiting PC-3 invasion, at least 500-fold more resistant to CYP24A1-dependent degradation and has about 25-fold lower binding affinity to DBP (Table 1). In addition, MART-10 did not raise serum calcium when it was injected into rats [D. Iglesias-Gato et al., unpublished observation]. The unique properties of MART-10 suggest that this analog has a potential as a new regimen for prostate cancer treatments through all stages of the disease.

## Figures and Tables

**Table 1:** Comparison of biological activity between 1*α*,25(OH)_2_D_3_ and MART-10 in prostate cancer cells.

	Anti-proliferation	Anti-invasion	CYP24A1, *K*_cat_/*K*_*m*_	DBP Binding
1*α*,25(OH)_2_D_3_	1	1	1	1
MART-10	1,000	100	1/500	1/25

## Abbreviations

1*α*,25(OH)_2_D_3_: 1*α*,25-dihydroxyvitamin D_3_
VDR: Vitamin D receptor
DBP: Vitamin D binding protein
MMP-9: Matrix metalloproteinase-9
O2C3: 2*α*-(3-hydroxypropoxy)-1*α*,25(OH)_2_D_3_
MART-10: 19-nor-2*α*-(3-hydroxypropyl)-1*α*,25(OH)_2_D_3_
MART-11: 19-nor-2*β*-(3-hydroxypropyl)-1*α*,25(OH)_2_D_3_.
